# eRegCom—Quality Improvement Dashboard for healthcare providers and Targeted Client Communication to pregnant women using data from an electronic health registry to improve attendance and quality of antenatal care: study protocol for a multi-arm cluster randomized trial

**DOI:** 10.1186/s13063-020-04980-1

**Published:** 2021-01-11

**Authors:** Kjersti Mørkrid, Binyam Bogale, Eatimad Abbas, Khadija Abu Khader, Itimad Abu Ward, Amjad Attalh, Tamara Awwad, Mohammad Baniode, Kimberly Suzanne Frost, Michael James Frost, Buthaina Ghanem, Taghreed Hijaz, Mervett Isbeih, Sally Issawi, Zaher A. S. Nazzal, Brian O’Donnell, Sharif E. Qaddomi, Yousef Rabah, Mahima Venkateswaran, J. Frederik Frøen

**Affiliations:** 1 Division for Health Services, Global Health Cluster, Norwegian Institute of Public Health, PB 222 Skøyen, 0213 Oslo, Norway; 2 Centre for Intervention Science in Maternal and Child Health, University of Bergen, Bergen, Norway; 3 Palestinian National Institute of Public Health, Ramallah, Palestine; 4 The Palestinian Ministry of Health, Ramallah, Palestine; 5 Institute of Community and Public Health, Birzeit University, Ramallah, Palestine; 6 Health Information Systems Programme, Department of Informatics, University of Oslo, Oslo, Norway; 7 Faculty of Medicine and Health Sciences, An-Najah National University, Nablus, Palestine

**Keywords:** Attendance, DHIS2, Quality of care, Antenatal care, Maternal and newborn health, eHealth, Digital health, Electronic registry, eRegistries, Health systems, SMS, Audit and feedback, Effective coverage, Targeted Client Communication, Palestine

## Abstract

**Background:**

This trial evaluates interventions that utilize data entered at point-of-care in the Palestinian maternal and child eRegistry to generate Quality Improvement Dashboards (QID) for healthcare providers and Targeted Client Communication (TCC) via short message service (SMS) to clients. The aim is to assess the effectiveness of the automated communication strategies from the eRegistry on improving attendance and quality of care for pregnant women.

**Methods:**

This four-arm cluster randomized controlled trial will be conducted in the West Bank and the Gaza Strip, Palestine, and includes 138 clusters (primary healthcare clinics) enrolling from 45 to 3000 pregnancies per year. The intervention tools are the QID and the TCC via SMS, automated from the eRegistry built on the District Health Information Software 2 (DHIS2) Tracker. The primary outcomes are appropriate screening and management of anemia, hypertension, and diabetes during pregnancy and timely attendance to antenatal care. Primary analysis, at the individual level taking the design effect of the clustering into account, will be done as intention-to-treat.

**Discussion:**

This trial, embedded in the implementation of the eRegistry in Palestine, will inform the use of digital health interventions as a health systems strengthening approach.

**Trial registration:**

ISRCTN Registry, ISRCTN10520687. Registered on 18 October 2018

## Background

Scale up of effective, high-quality interventions is essential in order to reach the Sustainable Development Goals and achieve Universal Health Coverage in maternal and child healthcare [1, 2]. Antenatal care (ANC), postpartum care (PPC), and newborn care in low- and middle-income countries show low effective coverage [3, 4].

Countries can improve healthcare systems by exploiting the potential of digital technology. Digital health information systems with individual-level data and mobile technologies are expanding globally and provide an opportunity to support, involve, and influence healthcare providers and their clients’ behavior [5–7]. However, effective digital health interventions are complex and require careful design, implementation, and evaluation [8].

Audit and feedback, widely used quality improvement interventions, allow healthcare providers to assess and adjust their performance and, as a result, improve the quality of the care they deliver [9, 10]. Audit and feedback can be defined as any summary of clinical performance of healthcare over a specified period of time and may include recommendations for clinical actions [11]. It is most effective when it is presented more than once, given both verbally and in writing, provided by a supervisor or respected colleague, presented with clear goals and action plans, aimed to decrease targeted behavior, directed towards the recipient with room for improvement, and to health professionals typically working with guideline-bound clinical activities [12]. The model of actionable feedback emphasizes that feedback should be timely, individualized, non-punitive, and meaningful to the recipient to be effective [13]. Explicit use of theory to inform intervention development, and user involvement in all stages of a digital health intervention development, implementation, and evaluation, is recommended, but seldom described [14–17].

Digital Targeted Client Communication (TCC) can utilize data, such as demographic characteristics and health status, to tailor communications to an individual’s specific needs if good-quality individual-level data are available [18, 19]. Short message service (text message (SMS)) is often used as a medium to deliver TCC interventions. Appointment reminders alone, or coupled with generic health promotion messages, can impact people’s knowledge, health literacy, and attitudes and thus improve healthy behavior and utilization of healthcare services [17, 20–23]. However, even though tailored, co-designed, theory-driven TCC interventions tend to be more effective than generic messages, evidence is needed, especially in the field of maternal and child health (MCH) in low- and middle-income countries [15, 19, 21, 22].

### Palestinian context

The total population in Palestine is 4.17 million, 2.58 million (62%) in the West Bank and 1.59 million (38%) in the Gaza Strip [24]. There are approximately 62,000 live births in the West Bank and 56,000 live births in the Gaza Strip annually. Individuals under 18 years of age constitute 48% of the total population, and the fertility rate is 3.8 in the West Bank and 4.9 in the Gaza Strip.

MCH is an important area in the Palestinian healthcare system, and gestational anemia, hypertensive disorders of pregnancy, gestational diabetes mellitus, and fetal growth restriction are identified as priority conditions for prevention [25]. Approximately 43% of pregnant women attend public ANC services, but only 13% according to the recommended national schedule, which results in low effective coverage [26]. Both the utilization and quality of health services have room for improvement. MCH supervisors, each responsible for 16 to 54 public primary healthcare clinics (PHCs), supervise midwives, nurses, and community health workers, mainly by checking registers and clinical equipment [27].

The routine governmental documentation tool for ANC and PPC in Palestine is the eRegistry built on the District Health Information Software 2 (DHIS2) Tracker [28]. The eRegistry is a web-based open-source information system for the longitudinal collection, storage, retrieval, analysis, and dissemination of information on health determinants and outcomes for individual persons [5, 29]. The implementation of the Palestinian eRegistry was carried out by the Ministry of Health (MoH) and the Palestinian National Institute of Public Health (PNIPH), with support from the Norwegian Institute of Public Health and the University of Oslo, as a measure to support healthcare providers in screening and management, and improve monitoring and evaluation [30, 31].

The eRegistry in Palestine provides clinical decision support based on national guidelines, including referral recommendations. It creates longitudinal pregnancy records and removes the need for secondary data reporting or manual aggregation for reporting. The eRegistry’s comprehensive information gathered in a continuous fashion allows TCC via SMS to clients and QID for healthcare providers, but the effectiveness is unknown.

### Objectives

The primary objectives of the eRegCom cluster randomized controlled trial (CRCT) are, compared to the basic eRegistry functionalities in public MCH PHCs in Palestine, to estimate the effectiveness of the eRegistry’s:

- Quality Improvement Dashboard (QID) on improving appropriate screening and management for anemia, hypertension, and diabetes during pregnancy by the healthcare provider
- Targeted Client Communication (TCC) on improving timely attendance to ANC by the woman
- QiD and TCC interventions combined on the measures described above

**Fig. 1.**
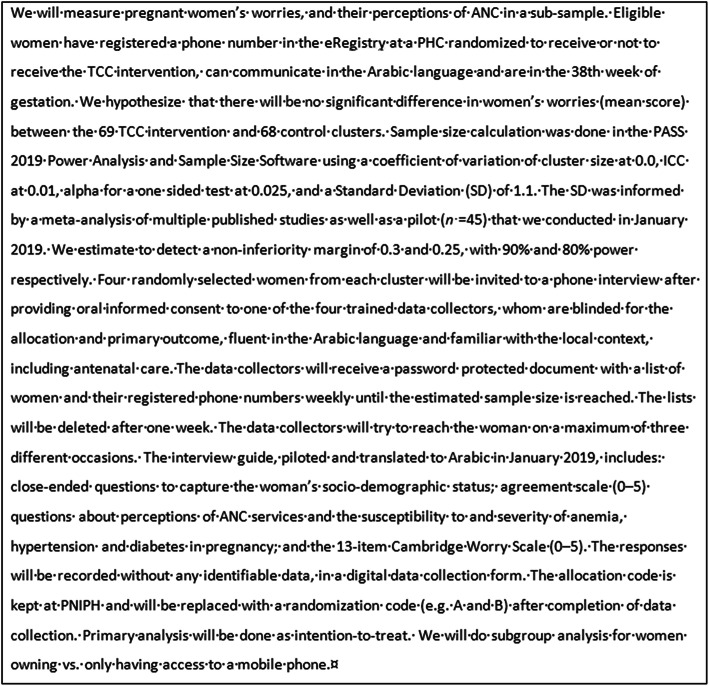
The effect of the TCC intervention on pregnant women’s worries in a sub-sample

We will also estimate the effectiveness across equity measures and, in a sub-sample, assess the effect of the TCC intervention on pregnant women’s worries and perceptions of ANC, including the quality of care and information received from the health system (Fig. 1).

## Methods

### Trial design

This CRCT is a superiority trial with four parallel arms (TCC, QID, TCC and QID, and control). The unit of randomization is an individual PHC, with the exception of two clusters. These two clusters include two PHCs each, where the pair of PHCs is served by the same healthcare provider.

### Study setting

The most common organizational structure in the public maternal and child healthcare system in Palestine includes two healthcare providers per clinic, mainly midwives, nurses, and/or community health workers, in addition to a doctor serving several clinics. Each user in the governmental eRegistry has a unique username and password, which enable access to records and specific system features according to their assigned role. The woman’s personal ID number is used to create an electronic ANC record for her pregnancy. The sociodemographic, obstetric, and medical information including clinical tests, laboratory, and ultrasound measurement results are entered into the eRegistry at point-of-care. The data trigger different digital health interventions such as the guideline-based clinical decision support and automated public health reports. Women identified with certain risk factors are referred for additional management, and their records are available in the so-called high-risk clinics after referral [31].

### Eligibility criteria

**Fig. 2:**
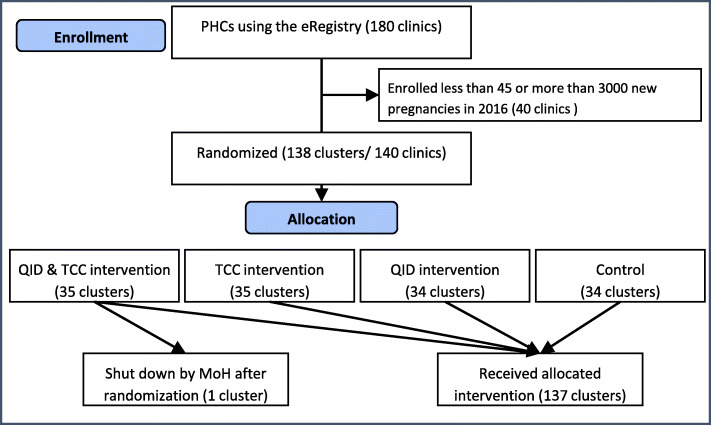
Flow diagram showing eligible primary healthcare clinics (PHCs) and allocation

All public PHCs offering ANC and PPC services using the eRegistry were eligible for the study. PHCs that enrolled less than 45 or more than 3000 new pregnancies in 2016 were excluded. No exclusion criteria were made based on individual healthcare provider’s or pregnant women’s characteristics (Fig. 2).

A total of 138 clusters (individual PHC and a combination of two PHCs served by the same healthcare provider for two clusters) were eligible for randomization. They enrolled a mean of 344 and a median of 131 new pregnant women in 2016 (Additional file 1). Data from all ANC visits occurring in the included PHCs during the study period will be used to assess our outcomes.

### Intervention

The interventions are at the cluster-level targeting nurses, midwives, and community health workers, referred to as healthcare providers, and their clients.

#### The Quality Improvement Dashboard intervention

The QID intervention is the provision of access to the QID within their routine eRegistry, as well as training of healthcare providers on how to use it.

As part of the QID intervention development process, public healthcare providers, supervisors, and health system administrators were interviewed to map the existing supervision and feedback system [27]. We found that healthcare providers received irregular supervision visits with limited focus on performance improvement at the clinical level. Findings and recommendations regarding benchmarking [32], the use of SMART (Specific, Measurable, Achievable, Relevant and Timely) criteria, concepts from social nudging and Enhanced active choice [33], and the Model of Actionable Feedback [13] informed the design of the QID. eRegistry users’, nursing and medical directors’, and PNIPH and MoH staff’s reviews informed the revisions. The final version was translated into the local Arabic language.

The training curriculum is founded on quality improvement theories and models such as the Plan, Do, Study, Act (PDSA) cycles [34]. Healthcare providers learn how they can use the QID, which presents indicators of quality gaps in their clinic, as a tool to improve the quality of the care they provide.

**Fig. 3:**
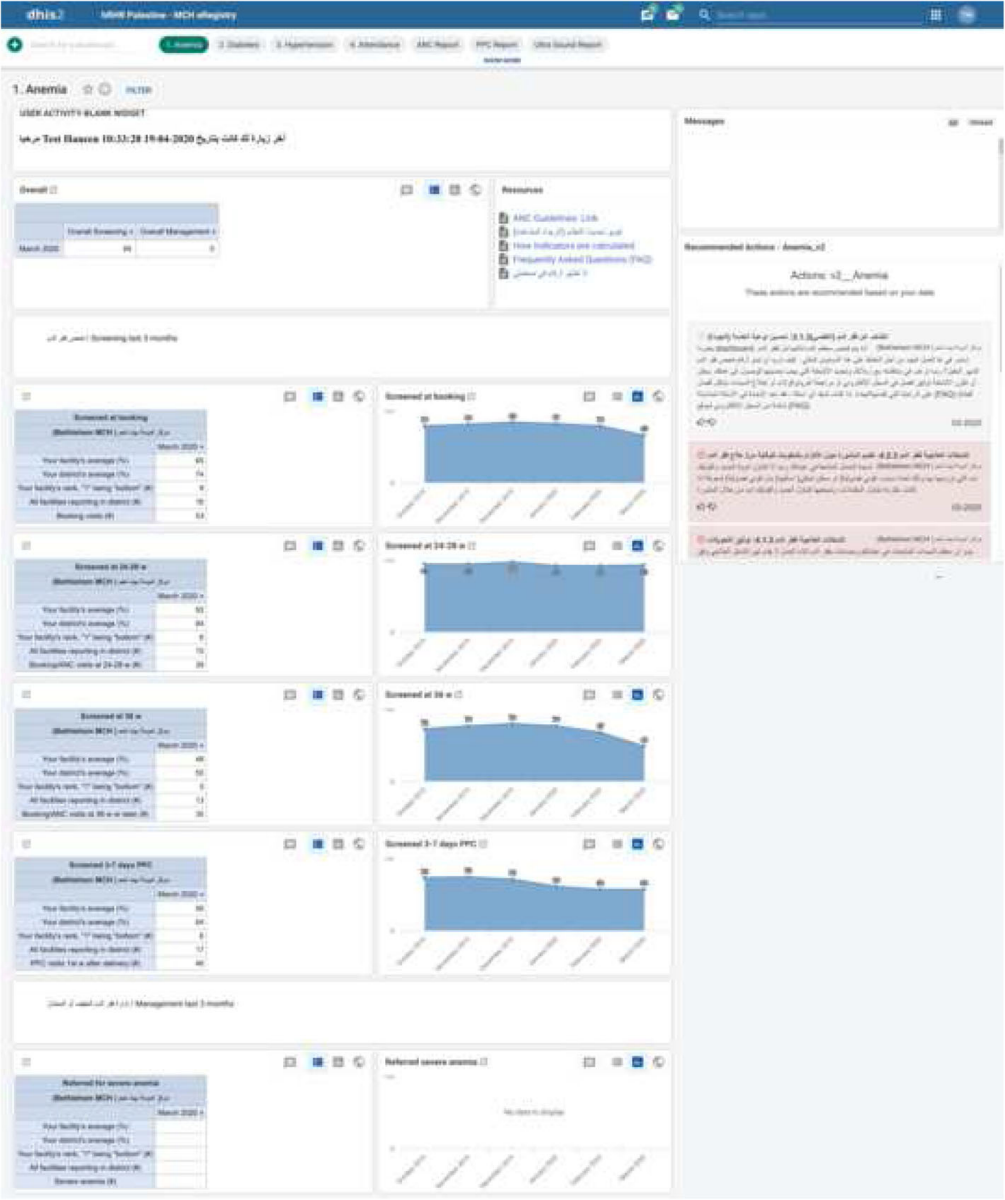
Quality Improvement Dashboard for anemia to healthcare providers from the eRegistry. Top left: individualized on clinic level; top right: reminders of the week’s focus area, namely anemia, hypertension, diabetes, or attendance; tables: indicators with benchmarks; graphs: percentage average over the last 3 months; right: action items presented in colors (green = good performance, yellow = room for improvement, red = large room for improvement)

The QID includes four tabs for each focus area, namely anemia, hypertension, diabetes, and attendance. Healthcare providers are given a new focus area every week through a message in the eRegistry that congratulates a good performing PHC or presents an evidence-based statement on the week’s focus area. Each tab contains performance indicators calculated from data entered at point-of-care. The indicators are presented as an average over the last 3 months, in both tables and graphs, and benchmarked with clinics within the district. The clinic’s performance level—defined as an index of the absolute and relative values of an overall screening and an overall management indicator—drives the appropriate action items for each focus area. Two screening and two management action items that include recommendations for improvement are presented in colors (green = good performance, yellow = room for improvement, red = large room for improvement) with monthly updates (Fig. 3). The healthcare providers can score the action items via a thumbs up/down icon and add written comments.

#### The Targeted Client Communication intervention

The TCC intervention is automated text messages sent via SMS from the eRegistry to pregnant women, and training of healthcare providers on how to enroll women in the program.

The TCC intervention development process, including the full-text message library, is described in detail elsewhere [35]. In short, pregnant women and healthcare providers were interviewed to identify how pregnant women perceive their risks of getting anemia, hypertension, and diabetes during pregnancy and susceptibility to fetal growth restriction, in addition to benefits and barriers of attending ANC, using the Health Belief Model. The findings, concepts from social nudging and Enhanced active choice [33], and the Model of Actionable Feedback [13] informed the content, medium, timing, and frequency of the TCC intervention.

The training includes how to register and withdraw women, and the timing and the content of the text messages. Healthcare providers register women that agree to receive text messages by ticking a box in the eRegistry. This can be done at any time in gestation, preferably at the first visit.

The tailored text messages include the woman’s name and her clinic’s name and may also include the date of her next appointment (Table 1). Those that include information about one or more of the high priority areas (anemia, hypertension, and diabetes during pregnancy, and fetal growth restriction) will be sent at the time these conditions are screened for, namely at the 16, 18–22, 24–28, 32, or 36 weeks’ gestation routine ANC visit. However, women with documented anemia, hypertension, diabetes, or fetal growth restriction will not receive the information about routine screening for that condition, e.g., women with diabetes will not receive a text message about the routine screening for diabetes at 24–28 weeks’ gestation. The text messages will be sent to the mobile number registered on the woman after working hours during ANC at the following time points:

- At registration, a welcome message including information about how to un-enroll
- One week prior to a timely scheduled routine visit
- Three days prior to a timely scheduled routine visit to women with risk factors for anemia, hypertension, diabetes, or fetal growth restriction
- Twenty-four hours prior to a visit
- Twenty-four hours after a missed timely scheduled routine visit
- Recapture message 24 h prior to the start of the appropriate time window for a routine visit to women without any timely scheduled routine visit in the future

**Table 1:** Example text messages send to an overweight pregnant woman without identified anemia, diabetes, or hypertension

Welcome	One week prior to a visit	Three days prior to a visit	Twenty-four hours prior to a visit	Twenty-four hours after a missed visit	Twenty-four hours prior to a missing visit
Dear Abi, Most women attend antenatal care for their own and baby’s health. The healthcare provider will measure your blood pressure, hemoglobin and blood glucose level. You will receive text message appointment reminders. Please let us know if you do not want these messages. Tamoon clinic	Dear Abi, The date of your upcoming appointment is 2020.04.20. One in 20 develop high blood pressure in pregnancy, and this may affect your health and the growth of your baby. The healthcare provider will measure your blood pressure and the amount of protein in urine as they may be a sign of high blood pressure. Tamoon clinic	Dear Abi, 2020.04.20 is your next appointment, as agreed. High body weight before pregnancy, may increase the risk of developing high blood pressure. The healthcare provider will measure your blood pressure and the amount of protein in urine at your next visit. Tamoon clinic	Dear Abi, This is a reminder that you have an appointment tomorrow, 2020.04.20, as agreed. Tamoon clinic	Dear Abi, Sorry to have missed you at yesterday’s appointment. We hope to see you back with us for antenatal care as soon as possible. Tamoon clinic	Dear Abi, You haven’t scheduled your next appointment, which is coming up soon. Timely attendance to every antenatal care visit may ensure you and your baby's safety, through early detection and treatment of complications. Please contact us to schedule your next visit. We are ready to provide you with care, and hope to see you soon. Tamoon clinic

#### Adherence, training, and concomitant care

The TCC and QID interventions in the eRegistry are available to healthcare providers working in PHCs randomized to trial arms receiving one or both of the digital health interventions. The research team trained the trainers, who were eRegistry staff at PNIPH and nursing directors, each representing a governorate. Healthcare providers from TCC and QID intervention PHCs were trained separately, and healthcare providers working in PHCs randomized to both interventions received both trainings. The trainings were conducted in the first quarter of 2019, and each session lasted for half a day. A follow-up training session was conducted in September 2019. Healthcare providers in QID intervention PHCs received a video presenting new functionalities in the QID at the start of the trial. New employees in intervention PHCs will receive on-site training from one of the trainers.

MCH supervisors will carry out similar periodic supervision visits to all PHCs in all arms of this CRCT. Women will not receive any concomitant care across intervention and control PHCs.

### Outcomes

The outcomes represent key areas of quality concerns in Palestine and are in line with our previous eRegQual CRCT, a study assessing the effect of using an eRegistry versus paper-based ANC records [25]. The Palestinian ANC guidelines for the recommended gestational week (Table 2) for screening and management of anemia, hypertension, and diabetes have defined our outcomes. For routine visits recommended for one specific week, we have made the time window for the outcome measures 2 weeks wider than the guideline, to allow some flexibility for maternal choice and time for e.g. laboratory results to be received and documented in the eRegistry.

**Table 2:** Time windows for the outcome measures and routine ANC visits including the primary screening test

	First visit	Routine visits				
**Recommended occurrence of ANC visits, GW**	Any week	16	18–22	24–28	32	36
**Included in outcome measures, GW**	Any week ± 7 days	15–17	18–22	24–28	31–33	35–37
**Primary screening tests for priority conditions**^a^	Blood pressure, hemoglobin, urine glucose	Blood pressure, fetal growth	Blood pressure	Blood pressure, hemoglobin, blood glucose	Blood pressure, fetal growth	Blood pressure, hemoglobin, fetal growth

*ANC* antenatal care, *GW* weeks of gestation

^a^Anemia, hypertension, diabetes, and fetal growth restriction according to the Palestinian guidelines

#### Primary outcomes

##### Comparison I: QID arm vs control arm

The primary outcome in comparison 1 is the proportion of women who receive appropriate screening and management of anemia, hypertension, and diabetes (Tables 3, 4, 5, 6, and 7). For example for anemia, the proportion is calculated by identifying the number of ANC visits where anemia should have been screened for and/or managed (denominator), and among them where anemia was appropriately screened for and/or managed (numerator). Only the first step in the management algorithm will be included (Tables 3, 4, 5, 6, and 7). Pregnant women with documented ongoing anemia, hypertension, or diabetes prior to the timely routine visit will not be included in the denominator for appropriate screening and management of anemia, hypertension, and diabetes respectively.

**Table 3:** Anemia screening test, result, and appropriate management

Hemoglobin test at first visit, 24–28, and 36 weeks’ gestation		
Hb ≥ 11 g/dL	Hb 10.9–7 g/dL	Hb < 7g/dL
No further action	Hb after 1 month	Refer to hospital

**Table 4:** Hypertension screening test, result, and appropriate management prior to 20 weeks’ gestation

Blood pressure prior to 20 weeks’ gestation, test at the first visit and at 16, 18–22 weeks’ gestation	
SBP < 140 mmHg and DBP < 90 mmHg	SBP ≥ 140 mmHg, DBP ≥ 90 mmHg
No further action	Refer to high-risk clinic

*SBP* systolic blood pressure, *DBP* diastolic blood pressure

**Table 5:** Hypertension screening test, result, and appropriate management at or after 20 weeks’ gestation

Blood pressure at or after 20 weeks’ gestation, test at 18–22, 24–28, and 36 weeks’ gestation		
SBP < 140 mmHg and DBP < 90 mmHg	DBP 90–99 mmHg, SBP 140–149 mmHg	DBP ≥ 100 mmHg, SBP ≥ 150 mmHg
No further action	New BP within 4 days	Refer to hospital

*SBP* systolic blood pressure, *DBP* diastolic blood pressure

**Table 6:** Diabetes screening test, result, and appropriate management prior to 24 weeks’ gestation

Urine glucose test at booking prior to 24 weeks’ gestation	
Negative	Positive
No further action	Blood glucose test

**Table 7:** Diabetes screening test, result, and appropriate management at or after 24 weeks’ gestation

Blood glucose test at 24–28 weeks’ gestation		
RBG < 105 mg/dL, FBG < 95 mg/dL	RBG 105–140 mg/dL, FBG 95–126 mg/dL	RBG ≥ 140 mg/dL, FBG ≥ 126 mg/dL
No further action	New blood test within 3 weeks	Refer to a diabetes clinic

*RBG* random blood glucose, *FBG* fasting blood glucose

##### Comparison II: TCC arm vs control arm

The primary outcome in comparison 2 is the proportion of all timely routine ANC visits that a woman was eligible for, where the woman attended (Table 2). The first ANC booking visit is excluded, and each timely routine visit will be counted separately as a singular opportunity to succeed or fail in attendance.

##### Comparison III: QID + TCC arm vs control arm

The primary outcome in comparison 3 is the product of the quality of care (comparison 1) and the utilization of care (comparison 2) to assess the effective coverage. For example for anemia, it is the proportion of all timely routine ANC visits where anemia should have been screened for and/or managed, where the woman attended and anemia was appropriately screened for and/or managed.

##### Effect on healthcare equity

We will assess the effects of the interventions on the primary outcomes across equity measures used in routine statistics in Palestine. The data points include average monthly household incomes (less than 200; 200–900; 901–1824; 1825–3054; and > 3055 Israeli new Shekel), mother’s years of education (< 10; 10–13 years; > 13 years), age at marriage (less than 20; 21–25; 26–30; 31–35; 36–40; greater than 40 years), and age at first pregnancy (less than 20; 20–25; 26–30; 31–35; 36–40; greater than 40 years).

##### Other outcomes

We will assess the proportion of women receiving appropriate screening and appropriate management separately at each visit for anemia, hypertension, and diabetes. We will also measure the retention rate from ANC to PPC and timely screening and management of fetal growth. The data entry in the eRegistry is continuous and the randomization permanent, which enable the analysis of overall health outcomes in a continuous and longitudinal manner for each individual woman. We might assess other outcomes if the intervention(s) shows an effect.

### Timeline

**Fig. 4:**
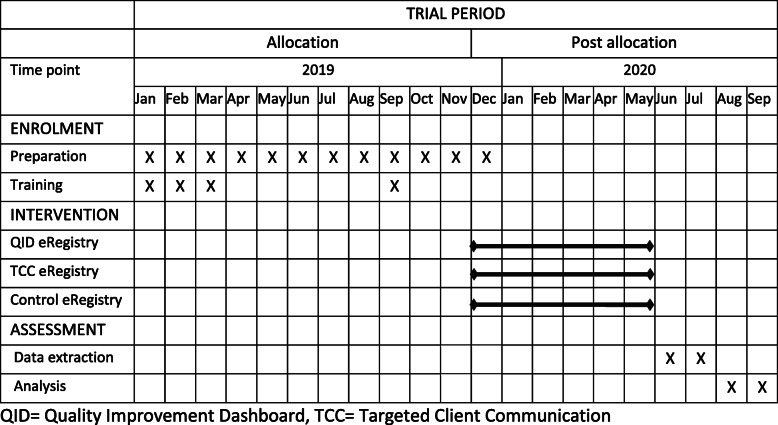
Schedule of enrolment, interventions, and assessments (SPIRIT figure)

Care providers in PHCs randomized to the TCC intervention have had the opportunity to register pregnant women to the text message service since June 2019. Care providers in PHC randomized to the QID intervention received access to the QID December 2019. We expect to reach the target sample size after approximately 6 months (Fig. 4).

### Sample size

Sample size calculations for the primary outcomes were performed in STATA “clustersampsi” (StataCorp. 2015. Stata Statistical Software: Release 14. College Station, TX: StataCorp LP). We estimated the minimum detectable difference for comparison of each intervention arm with the control arm without assuming the effect of multiple comparison (using a single control to each intervention arm). The control prevalence for the primary outcome, using data from the eRegQual CRCT, ranged from 30 to 85% for comparison I (QID vs control) and from 34 to 53% for comparison II (TCC vs control) (unpublished results). The 138 eligible clusters have an average of 172 new pregnancies per 6 months (ranging from 23 to 1500) and a 1.69 coefficient of variation of cluster size (2016 data). We assumed a priori intra-cluster correlation coefficient (ICC) of 0.05 and set the statistical significance level to 5%. We are 80% powered to detect an estimated relative 50% increase for the outcomes with the smallest control prevalence (e.g., improving anemia screening and management at 24–28 weeks’ gestation from 30 to 45%).

### Recruitment

PHCs that fulfilled the recruitment criteria were randomized to one of the four arms. Healthcare providers working in PHCs randomized to one or both of the interventions received training as described (the “Intervention” section).

No financial or non-financial incentives are or will be provided to the woman, public health officers, or healthcare providers at the PHCs included in the trial.

### Allocation

Statisticians at the Center for Intervention Science in Maternal and Child Health (CISMAC), University of Bergen, Norway, performed the randomization independently without any influence from the research team.

PHCs were allocated to the TCC intervention, QID intervention, TCC and QID intervention, or control group with an equally 1:1:1:1 randomization ratio. The randomization was stratified by the point in time the eRegistry was implemented, and constrained on laboratory availability, ultrasound availability, and the size of the PHC.

In total, 10,000 randomization allocations were generated. The 10% best and balanced allocations with the least differences between the arms for the given covariates were identified, and one of these allocations were randomly selected for the trial [36].

### Blinding

Healthcare providers and women attending care at PHCs included in the trial are blinded to the outcome measures. Women are expected to be blinded to the allocation of the QID intervention, but not to the TCC intervention due to the nature of the intervention. Healthcare providers are not blinded to the allocation of any intervention.

Data will be extracted from the eRegistry and transformed to anonymous datasets by blinded data analysts at PNIPH. Allocation codes will be generated for each primary outcome separately (e.g., A, B, C, and D; E, F, G, and H; I, J, K, L, and M). Our independent trial monitors (CISMAC) will keep the codes, which will be provided as allocation groups (intervention TCC, intervention QID, intervention TCC and QID vs control) for each set of outcomes after the completion of analyses.

### Data extraction methods

Healthcare providers will routinely and continuously enter data in the eRegistry during patient care, and all outcomes are informed by secondary data. Anonymous data will be extracted from the eRegistry in accordance with the standard operating procedure (SOP) of the Palestinian maternal and child health eRegistry for routine registry operations and use of data for research purposes.

### Data management

The data in the eRegistry will be managed in accordance with the governance structure approved by the Palestinian MoH. Only pre-defined anonymous data needed for the outcomes will be extracted from the eRegistry by PNIPH staff for this trial. The pre-defined anonymous original dataset will be provided to the trial sponsors and to our independent trial monitors (CISMAC), for independent monitoring and safeguarding.

### Statistical methods

Intention-to-treat analysis will be performed for the primary outcomes to compare each independent intervention arms (QID, TCC, and QID and TCC) with the control, using individual-level data taking the design effect of the clustering into account. Descriptive statistics will be reported, and appropriate tests will be used to compare variables between the groups. Statistical significance will be set at *p* < 0.05. Appropriate bivariate and multivariate regression analyses will be performed. The regression analyses will take the design effect of the clustering into account and enable adjustment for any relevant variables not accounted for during randomization. We will do complete case analyses and consider appropriate imputations for missing data. We will present cluster-wise effects of the intervention to explore whether a disproportionately large part of the effect can be ascribed to extreme effects in a few large clusters. The interaction effect of the two interventions will be performed as a secondary analysis. We will conduct per-protocol analyses in cases of protocol violations, including withdrawal of the eRegistry, and data from these clusters will be excluded from the time of violation when appropriate. Data will be analyzed using the latest version of STATA (Stata Statistical Software: College Station, TX: StataCorp LP).

## Data monitoring

Data management and monitoring will be done in accordance with the SOP of the Palestinian maternal and child health eRegistry for routine registry operations. We have not designated a Data and Safety Monitoring Committee, due to the lack of potential significant harm, nor an Endpoint Adjudication Committee, since we will not use any subjective clinical data for outcome measures.

### Harms

This CRCT only utilizes the moment of opportunity of an ongoing implementation to study new digital interventions in a health systems approach. No potential for clinically significant harm has been identified during the development and implementation of this trial. Potential discomfort from worries may occur among women attending ANC in clusters receiving the TCC intervention, and this will be examined in a sub-sample (Fig. 1).

### Confidentiality

Data confidentiality will be handled in accordance with the Palestinian MoH’s legal framework for maternal and child health electronic registries. This CRCT will only utilize anonymous data to enable the assessment of the effectiveness of the interventions. We will publish only aggregate data, and no data on individual clients, care providers, or identifiable clusters will be published.

### Access to data

The data in the eRegistry belong to the Palestinian MoH, and the researchers will not have access to the entire registry or identifiable data of any kind. We will not publish the full data set as our legal rights to the data is limited to this analysis, and the richness of data would allow for several other analyses for which there is no ethics approval or approval from the Palestinian MoH. We will, however, publish syntaxes needed to recreate the data set from the eRegistry.

### Dissemination plan

This protocol follows the Standard Protocol Items: Recommendations for Intervention trials (SPIRIT) guidelines. A formal revision of the protocol will be done if the change can affect the study’s nature. The sponsors and our independent trial monitors (CISMAC) will have to agree on the revision, which will require renewed approvals from both the ethic committee in Norway and in Palestine, and the Palestinian health authorities. We will inform all users and stakeholders and publish the results of the CRCT in peer-reviewed open-access journals according to the Consolidating Standards of Reporting Trials (CONSORT) guidelines and the mHealth Evidence Reporting and Assessment (mERA) checklist. Results will also be presented at scientific meetings and congresses and to the Palestinian MoH directly. With permission from the MoH, we will inform all participating PHC and their staff directly. We will acknowledge any change in the study outcomes, study design, sample sizes, or significant administrative aspects that will impact the study’s nature when disseminating the findings. Authorship will be in line with the recommendations of the International Committee of Medical Journal Editors. Summaries of the results and other relevant information will be published on the eRegistries website.

## Discussion

We have designed a multi-arm CRCT with two digital health interventions, namely the QID for healthcare providers and TCC sent via SMS to pregnant women. The eRegistry employs the collection and use of systematic, uniform, and longitudinally entered routine clinical data, in algorithms that instantly drive these digital health interventions [5]. The QID and TCC interventions’ can impact the quality of the ANC service and the utilization of care by the pregnant woman, and the eRegCom CRCT aims to estimate the effectiveness.

Routine data from the eRegistry in Palestine demonstrates that both quality of care and utilization of care have significant room for improvement, and both need to be addressed to achieve a high effective coverage. To ensure healthy lives and promote well-being for all at all ages (Sustainable Development Goal 3), data and digital health are highlighted as accelerators [37]. Our interventions directly address the traditional lack of available clinical data in a timely manner from routine health information system to healthcare providers and clients in low- and middle-income countries [38], and the trial is a direct response to the WHO review group on digital health interventions’ request for effectiveness studies [19].

We used the Principles for Digital Development [39] and a human-centered design approach [40] to develop the eRegistry, and also the QID and TCC interventions. The Palestinian MoH and PNIPH have been heavily involved in all stages of the CRCT and led the communication towards healthcare providers. We will, in addition, assess the effect of the TCC intervention on women’s worries in pregnancy and their perceptions of ANC, to ensure that we do not introduce harm. Also, the unintentional exacerbation of inequities based on owning versus having access to a mobile phone will be explored. We do not have data to explore inequities towards those that do not own or have access to a mobile phone, but acknowledge this issue.

Our experience from the pilot data collection and a recent time-motion study [41] is that women are willing to participate, and we expect a high response rate in the assessment of women’s worries and perception of ANC. We anticipate to follow a comprehensive sample of women throughout ANC due to Palestinian’s restrictions on movement. Our large sample of PHCs, with no individual-level eligibility criteria, is representative for Palestine and presumably also for other settings with a similar organizational structure.

One or two healthcare providers per PHC is the most common organizational structure in Palestine, but some healthcare providers may work or be relievers in more than one clinic. Healthcare providers working in both TCC intervention and control PHCs will receive the TCC training, but they can only register women to receive text messages from the TCC intervention PHC. Healthcare providers working in both QID intervention and control PHCs will not receive the QID training, and therefore not the full intervention. This is to avoid contamination, as the action needed to improve care can be done from any PHC without the QID tool itself. Even though very few PHCs in the trial are operated by the same healthcare provider, this may potentially lead to an underestimation of the effectiveness.

Other limitations of this trial include the cadres of healthcare professions we target, which is only nurses, midwives, and community health workers. We exclude doctors, due to the risk of contamination as they work in several PHCs. However, we acknowledge that doctors play a significant role in the care for pregnant women. Our outcomes are dependent on the completeness of documentation by the healthcare provider, as our data source, the eRegistry, is the routine governmental documentation tool with few required data points. In order for the clusters to have comparable opportunities to succeed or fail, when it comes to management, we only include the first step in the management algorithm for our primary outcomes. For example, women with mild and moderate anemia, a new hemoglobin result after 4 weeks, will be counted as a success, without taking into account the response to that result. However, we will assess the total management chain in the secondary analysis.

We work closely with the Palestinian MoH to mitigate the risk of changes to the PHCs’ activities and staff during the trial. A key aspect of the work with eRegistries is to facilitate the uptake of the evidence-based findings in other countries and promote our digital interventions as “Global Public Goods” [29]. The eRegistry does involve high start-up costs, but once implemented, the intervention only requires moderate additional investments. We are in the midst of a digital revolution, and more than 68 countries are using DHIS2 [28], the eRegistry’s platform. The added value of the digital interventions can add value to investments in eRegistries.

## Trial status

The CRCT began recruiting on 1 December 2019, and we expect to reach the target sample size after 6 months, approximately 1 June 2020. Protocol version 2—April 2020.

## Supplementary Information

**Additional file 1.** List of participating clinics and allocation

**Additional file 2.** Oral informed consent and interview guide

## Data Availability

Not applicable.

## Abbreviations

ANC: Antenatal Care
CISMAC: Center for Intervention Science in Maternal and Child Health
CRCT: Cluster Randomized Controlled Trial
DHIS2: District Health Information Software 2
MCH: Maternal and Child Health
MoH: Ministry of Health
NGO: Non-Governmental Organizations
PHC: Primary Healthcare Clinic
PNIPH: Palestinian National Institute of Public Health
PPC: Postpartum Care
QID: Quality Improvement Dashboard
SOP: Standard Operating Procedure
TCC: Targeted Client Communication
