# Supplementary material for: eRegCom—Quality Improvement Dashboard for healthcare providers and Targeted Client Communication to pregnant women using data from an electronic health registry to improve attendance and quality of antenatal care: study protocol for a multi-arm cluster randomized trial
Source: Trials. 2021 Jan 11;22:47. doi: 10.1186/s13063-020-04980-1 (PMC7802344; doi:10.1186/s13063-020-04980-1)
Supplement: Supplementary file 1 — Additional file 1. List of participating clinics and allocation [file 13063_2020_4980_MOESM1_ESM.pdf]

No.	Implementation Phase	Health District	PHCs (Clusters)	Cluster Size	Lab	UL	Intervention arm
1	Phase 2 the Gaza Strip	South	Absaan Al Jadejah	704	Yes	Yes	TCC_only
2	Phase 2 the Gaza Strip	Gaza	Al Daraj	2289	Yes	Yes	TCC_only
3	Phase 2 the West Bank	Qalqiliya	Al-gharbieh PHC	258	Yes	Yes	TCC_only
4	Phase 1 the West Bank	Bethlehem	Al-khader	56	Yes	Yes	TCC_only
5	Phase 2 the West Bank	Hebron	Al-Manshar	198	Yes	Yes	TCC_only
6	Phase 2 the West Bank	Qalqiliya	Al-markazyeh(central)	233	Yes	Yes	TCC_only
7	Phase 2 the West Bank	Jerusalem	Al-ram	56	Yes	Yes	TCC_only
8	Phase 2 the West Bank	North Hebron	Arabeyeh + Bani Na'im	396	No	No	TCC_only
9	Phase 2 the West Bank	Tulkarm	Attil	258	Yes	Yes	TCC_only
10	Phase 1 the West Bank	Nablus	Awarta	123	No	Yes	TCC_only
11	Phase 2 the Gaza Strip	South	Bani Sohelah	2674	Yes	Yes	TCC_only
12	Phase 1 the West Bank	Nablus	Bazria	94	No	Yes	TCC_only
13	Phase 2 the West Bank	Tulkarm	Beit Leed	105	Yes	Yes	TCC_only
14	Phase 2 the West Bank	South Hebron	Beit Awa	165	Yes	Yes	TCC_only
15	Phase 1 the West Bank	Ramallah&Al-bireh	Beit rema	140	Yes	Yes	TCC_only
16	Phase 1 the West Bank	Ramallah&Al-bireh	Beit Sera	47	Yes	Yes	TCC_only
17	Phase 2 the West Bank	Hebron	Ein Sarah	128	No	Yes	TCC_only
18	Phase 2 the West Bank	Qalqiliya	Hajah	101	Yes	Yes	TCC_only
19	Phase 1 the West Bank	Nablus	Hewarah	51	No	Yes	TCC_only
20	Phase 1 the West Bank	Jenin	Jalqamoos	69	Yes	No	TCC_only
21	Phase 2 the Gaza Strip	South	Joret Al loot	1205	Yes	Yes	TCC_only
22	Phase 2 the West Bank	Tulkarm	Kafr El-labad	102	No	Yes	TCC_only
23	Phase 2 the West Bank	Tulkarm	Kafr Jammal	85	Yes	Yes	TCC_only
24	Phase 1 the West Bank	Jenin	Meithalun	235	Yes	Yes	TCC_only
25	Phase 2 the West Bank	Tulkarm	Qafeen	241	Yes	Yes	TCC_only
26	Phase 1 the West Bank	Jenin	Raba	74	No	No	TCC_only
27	Phase 1 the West Bank	Ramallah&Al-bireh	Ramallah New MCH clinic	400	Yes	Yes	TCC_only
28	Phase 1 the West Bank	Nablus	Rojeeb	99	No	Yes	TCC_only
29	Phase 2 the West Bank	Yatta	Roqe't Al-Aroos	157	Yes	Yes	TCC_only
30	Phase 2 the West Bank	Tulkarm	Shwekeh	144	Yes	Yes	TCC_only
31	Phase 2 the West Bank	North Hebron	Surif Musqat + Beit Ummar	375	Yes	Yes	TCC_only
32	Phase 1 the West Bank	Nablus	Talfeet	116	Yes	Yes	TCC_only
33	Phase 2 the West Bank	Tubas	Tamoon Al-wosta	190	Yes	Yes	TCC_only
34	Phase 2 the West Bank	Hebron	Tarqomia	174	Yes	Yes	TCC_only
35	Phase 1 the West Bank	Nablus	Tel	109	Yes	Yes	TCC_only
36	Phase 2 the Gaza Strip	Gaza	Al Falah	2842	Yes	Yes	Control
37	Phase 2 the West Bank	Hebron	Al-haram	137	No	Yes	Control
38	Phase 2 the West Bank	Yatta	Al-karmel	95	Yes	Yes	Control
39	Phase 2 the West Bank	Hebron	Al-Rama	406	Yes	Yes	Control
40	Phase 2 the West Bank	South Hebron	Al-reheieh	166	Yes	Yes	Control
41	Phase 2 the West Bank	South Hebron	AL-Samooa'	167	Yes	Yes	Control
42	Phase 2 the West Bank	South Hebron	Al-thahreyeh	240	Yes	Yes	Control
43	Phase 1 the West Bank	Jenin	Al-zbabdeh	64	Yes	No	Control
44	Phase 2 the West Bank	Tulkarm	Anabta	124	Yes	Yes	Control
45	Phase 1 the West Bank	Jenin	Aneen	75	No	No	Control
46	Phase 2 the West Bank	Tubas	Aqaba	245	Yes	Yes	Control
47	Phase 1 the West Bank	Nablus	Beit Eba	69	No	Yes	Control
48	Phase 2 the West Bank	Hebron	Beit kahel	250	Yes	Yes	Control

49	Phase 2 the West Bank	North Hebron	Beit Ula	158	Yes	Yes	Control
50	Phase 1 the West Bank	Ramallah&Al-bireh	Bill'in	44	Yes	No	Control
51	Phase 1 the West Bank	Nablus	Central MCH clinic	1916	No	Yes	Control
52	Phase 1 the West Bank	Ramallah&Al-bireh	Ebween	52	Yes	Yes	Control
53	Phase 2 the West Bank	Hebron	Ethna	270	Yes	Yes	Control
54	Phase 2 the West Bank	Tulkarm	Far'oon	56	No	Yes	Control
55	Phase 1 the West Bank	Salfit	Health Directorate Salfit	486	Yes	Yes	Control
56	Phase 2 the West Bank	Jericho	Jericho MCH clinic	198	No	Yes	Control
57	Phase 1 the West Bank	Salfit	Kefel Hares	86	Yes	Yes	Control
58	Phase 2 the West Bank	North Hebron	Kfan Khamees	105	No	Yes	Control
59	Phase 2 the West Bank	North Hebron	Kharas	80	Yes	Yes	Control
60	Phase 1 the West Bank	Ramallah&Al-bireh	Kharbatha Bani Hareth	55	No	No	Control
61	Phase 1 the West Bank	Jenin	Misilyah	72	No	No	Control
62	Phase 1 the West Bank	Ramallah&Al-bireh	Nea'leen	109	Yes	Yes	Control
63	Phase 1 the West Bank	Nablus	Qabalan	184	Yes	Yes	Control
64	Phase 2 the Gaza Strip	South	Rafah	2290	Yes	Yes	Control
65	Phase 2 the West Bank	Yatta	Roqa'h	134	No	Yes	Control
66	Phase 2 the West Bank	Qalqiliya	Sanniriya	111	Yes	Yes	Control
67	Phase 2 the West Bank	Tubas	Tubas Central MCH clinic	204	Yes	Yes	Control
68	Phase 2 the Gaza Strip	Middle	Wadi Al Salqa	507	Yes	Yes	Control
69	Phase 1 the West Bank	Salfit	Yasoof and Izkaka clinic	92	Yes	Yes	Control
70	Phase 2 the Gaza Strip	Gaza	Al Salam	1756	Yes	Yes	QID_only
71	Phase 2 the Gaza Strip	Middle	Al Zawida	812	Yes	Yes	QID_only
72	Phase 1 the West Bank	Ramallah&Al-bireh	Al-Bireh MCH clinic	288	No	Yes	QID_only
73	Phase 2 the West Bank	Yatta	Al-derat	49	No	No	QID_only
74	Phase 2 the West Bank	Jerusalem	Al-Eizariya MCH clinic	347	Yes	Yes	QID_only
75	Phase 1 the West Bank	Jenin	Al-Fandaqumiya	89	No	No	QID_only
76	Phase 2 the West Bank	Yatta	Al-ghwetah	159	Yes	Yes	QID_only
77	Phase 2 the West Bank	Jericho	Al-ojah	81	Yes	Yes	QID_only
78	Phase 2 the West Bank	North Hebron	Al-Shuyukh	193	Yes	Yes	QID_only
79	Phase 2 the West Bank	South Hebron	Al-taqwa	112	Yes	Yes	QID_only
80	Phase 2 the Gaza Strip	Gaza	Atah Habeeb	2869	Yes	Yes	QID_only
81	Phase 2 the West Bank	Qalqiliya	Azoon	168	Yes	Yes	QID_only
82	Phase 2 the West Bank	Tulkarm	Bal'a	200	Yes	Yes	QID_only
83	Phase 1 the West Bank	Salfit	Bedia clinic	128	Yes	Yes	QID_only
84	Phase 1 the West Bank	Ramallah&Al-bireh	Beituniya	52	No	Yes	QID_only
85	Phase 1 the West Bank	Ramallah&Al-bireh	Deir Jarir	63	Yes	Yes	QID_only
86	Phase 2 the West Bank	South Hebron	Deir Samet Al-sharqyeh	90	Yes	Yes	QID_only
87	Phase 2 the West Bank	Qalqiliya	Hableh	202	Yes	Yes	QID_only
88	Phase 2 the West Bank	Jerusalem	Hezma	48	Yes	No	QID_only
89	Phase 1 the West Bank	Jenin	Jenin central clinic	1376	Yes	Yes	QID_only
90	Phase 2 the West Bank	Qalqiliya	kafr theleth	122	Yes	Yes	QID_only
91	Phase 1 the West Bank	Ramallah&Al-bireh	Kharbatha Al-mesbah	118	Yes	Yes	QID_only
92	Phase 2 the West Bank	Yatta	khelet al-Myeh	83	No	Yes	QID_only
93	Phase 1 the West Bank	Salfit	Kufr Al-deek	91	Yes	Yes	QID_only
94	Phase 2 the West Bank	Qalqiliya	kufr kadoom	59	Yes	No	QID_only
95	Phase 1 the West Bank	Jenin	Mirka	67	No	No	QID_only
96	Phase 1 the West Bank	Salfit	Qarawah	163	Yes	Yes	QID_only
97	Phase 2 the West Bank	North Hebron	Sa'eer	372	Yes	Yes	QID_only
98	Phase 1 the West Bank	Nablus	Salem	97	Yes	Yes	QID_only

99	Phase 2 the Gaza Strip	Gaza	Shek Radwan Clinic	1521	Yes	Yes	QID_only
100	Phase 1 the West Bank	Jenin	Silat adh Dhahr	337	Yes	Yes	QID_only
101	Phase 2 the West Bank	Hebron	Tafouh	259	Yes	Yes	QID_only
102	Phase 2 the West Bank	Hebron	Wad Azez	99	No	Yes	QID_only
103	Phase 2 the West Bank	Tulkarm	Zeita	62	Yes	Yes	QID_only
104	Phase 2 the Gaza Strip	South	Abasan Al Kabeerah	2555	Yes	Yes	TCC and QID
105	Phase 2 the Gaza Strip	Gaza	Abu Shebak	304	Yes	Yes	TCC and QID
106	Phase 2 the Gaza Strip	Gaza	Al Horyah	1408	Yes	Yes	TCC and QID
107	Phase 1 the West Bank	Bethlehem	Al-eabedieh	335	Yes	Yes	TCC and QID
108	Phase 2 the West Bank	Tubas	Alfara'h	85	Yes	Yes	TCC and QID
109	Phase 2 the West Bank	Hebron	Al-krantena	448	Yes	Yes	TCC and QID
110	Phase 2 the West Bank	Hebron	Al-masharqah	122	No	Yes	TCC and QID
111	Phase 2 the West Bank	Yatta	AL-mkabieh*	53	No	No	TCC and QID
112	Phase 2 the West Bank	Tulkarm	Al-reayeh Al-Janobeyeh	571	Yes	Yes	TCC and QID
113	Phase 1 the West Bank	Jenin	Al-Taibeh	57	No	Yes	TCC and QID
114	Phase 2 the West Bank	South Hebron	Al-thahreyeh Hosp. Emerg. & Delivery	411	Yes	Yes	TCC and QID
115	Phase 2 the West Bank	Tulkarm	Baqa ash Sharqiya	98	Yes	Yes	TCC and QID
116	Phase 1 the West Bank	Bethlehem	Bethlehem MCH clinic	229	Yes	Yes	TCC and QID
117	Phase 1 the West Bank	Nablus	Bieta	227	Yes	Yes	TCC and QID
118	Phase 1 the West Bank	Nablus	Borqah	65	No	Yes	TCC and QID
119	Phase 2 the West Bank	Tulkarm	Deir Al-ghsoon	213	Yes	Yes	TCC and QID
120	Phase 1 the West Bank	Ramallah&Al-bireh	Deir Amar	73	Yes	Yes	TCC and QID
121	Phase 1 the West Bank	Nablus	Doma	86	No	Yes	TCC and QID
122	Phase 2 the West Bank	South Hebron	Dora	303	Yes	Yes	TCC and QID
123	Phase 2 the West Bank	Qalqiliya	Emateen	51	Yes	Yes	TCC and QID
124	Phase 2 the West Bank	North Hebron	Halhol	214	Yes	Yes	TCC and QID
125	Phase 2 the West Bank	Tulkarm	Illar	164	Yes	Yes	TCC and QID
126	Phase 2 the West Bank	Qalqiliya	Jayous	90	Yes	Yes	TCC and QID
127	Phase 1 the West Bank	Jenin	Kferet	70	No	No	TCC and QID
128	Phase 1 the West Bank	Jenin	Kufr Raa'e	120	Yes	No	TCC and QID
129	Phase 2 the West Bank	Tubas	Old Tubas MCH clinic	229	Yes	Yes	TCC and QID
130	Phase 1 the West Bank	Ramallah&Al-bireh	Qarawah Bani Zeid	51	No	No	TCC and QID
131	Phase 1 the West Bank	Nablus	Ras Al-een	188	No	Yes	TCC and QID
132	Phase 1 the West Bank	Salfit	Sartah	111	No	Yes	TCC and QID
133	Phase 2 the West Bank	Tulkarm	Seda	109	No	Yes	TCC and QID
134	Phase 2 the West Bank	Yatta	Shoda Yata	53	No	No	TCC and QID
135	Phase 1 the West Bank	Ramallah&Al-bireh	Shuqba	60	Yes	Yes	TCC and QID
136	Phase 2 the Gaza Strip	South	Tal Sultan	1734	Yes	Yes	TCC and QID
137	Phase 2 the West Bank	Tulkarm	Thenabeh	70	Yes	Yes	TCC and QID
138	Phase 2 the West Bank	Tubas	Tyaseer	82	Yes	Yes	TCC and QID

* closed after randomization

UL=Ultrasound, Lab=laboratory

TCC=Targeted Client Communication, QID=Quality Improvement Dashboard
