# Supplementary material for: eRegCom—Quality Improvement Dashboard for healthcare providers and Targeted Client Communication to pregnant women using data from an electronic health registry to improve attendance and quality of antenatal care: study protocol for a multi-arm cluster randomized trial
Source: Trials. 2021 Jan 11;22:47. doi: 10.1186/s13063-020-04980-1 (PMC7802344; doi:10.1186/s13063-020-04980-1)
Supplement: Supplementary file 2 — Additional file 2. Oral informed consent and interview guide [file 13063_2020_4980_MOESM2_ESM.pdf]

Introduction:

1. Staff presents initial greetings, presents herself and that she calls on behalf of the Palestinian National Institute of Public Health, and seeks confirmation that the correct respondent is on the line.
2. Staff presents the purpose of the call and confirmation of an appropriate time: *I am calling you because we are doing research to improve the quality of antenatal care in public Primary Health Clinics. To inform this research, we are asking pregnant women some questions about their experience with the care and information they have received in clinics, and if they have any concerns or worries in pregnancy. Do you have a minute to let me explain how we found you and what we are inviting you to?*
    i. *If she indicates interest but lack of time/bad timing, it is allowable to suggest you call her back at a time suggested by her. You should not insist, and if she does not have time to respond at the subsequent planned call either, she should be considered as not consenting.*
3. Staff presents the mode of recruitment, the limits of information provided to her, and gets confirmation of pregnancy: *You have been selected randomly among women who are registered as pregnant by your care provider, and that has been reported to us, but I have no other information about you than your name and phone number. Is it correct that you are in the 9th month of your pregnancy? If she delivered, congratulate her, or if the pregnancy ended up in miscarriage, say sorry and thank for taking the call.*
4. Staff presents the content, and voluntary and confidential nature of the survey, ethics approval, as well as any potential benefit or harms, before seeking consent:
    a. *I would very much like to ask you some questions about your experience with antenatal care, the information you have received, and any worries you have*
    b. *It is voluntary for you to participate, and you can end the interview at any time you like, and you can decline to answer any question you want.*
    c. *Whether you chose to answer my questions or not will have no impact on the care you receive, and we will not share your answers with your care provider.*
    d. *You do not need to say anything about your health to answer the questions. It will only be yes and no questions, and some statements. I will only note your answers on a blank form without your name, phone number or any other identifiers on it, so nobody else than me will know that these are your answers.*
    e. *Our research has been approved by ethics committees, and we are obliged to preserve confidentiality.*
    f. *I expect that the questions will take about 10 to 15 minutes in total.*
    g. *Are you willing to participate by answering my questions?*
        i. *If now she indicates willingness but lack of time/bad timing, it is allowable to suggest you call her back at a time suggested by her. You should not insist, and if she does not have time to respond at the subsequent planned call either, she should be considered as not consenting.*

Demographics:

First, for us to understand why different women have different experiences, I would like to note a few things about you:

1. What is the name of your home district?
2. How many years of schooling have you completed?
3. Do you work/study outside your home?
4. Which year are you born?
5. Have you delivered a baby before?
6. In your previous pregnancy, have you ever encountered any adverse pregnancy outcomes (such as abortion, stillbirth, pre-term birth, or infant death)?
7. In this pregnancy, what was your gestational age at booking?
8. In this pregnancy, have you attended ultrasound in other clinics than your public primary healthcare clinic?
9. In this pregnancy, have you attended comprehensive ANC in other clinics than your public primary healthcare clinic?
10. In this pregnancy, have you been referred to a high risk clinic/specialist?

Susceptibility, Severity, Benefit:

Thank you, now I have some statements about antenatal care in general. I will present the statements, and would like to hear how much you agree to this, from 0 if you strongly disagree to 5 if you strongly agree. In between you have 1 If you moderately disagree, 2 if you slightly disagree, 3 if you slightly agree and 4 if you moderately agree. I can repeat the scale for you at any point.

11. It was important for me to attend all the scheduled antenatal care visits
12. The reason for why I chose to attend ANC was to be tested for diabetes
13. The reason for why I chose to attend ANC was to be tested for anemia
14. The reason for why I chose to attend ANC was to be tested for hypertension
15. The reason for why I chose to attend ANC was to have my baby's growth measured

Satisfaction, Public antenatal care clinic:

Thank you, now I have some statements about your experience with your public antenatal care clinic. I will present the statements, and would like to hear how much you agree to this, from 0 if you strongly disagree to 5 if you strongly agree. In between you have 1 If you moderately disagree, 2 if you slightly disagree, 3 if you slightly agree and 4 if you moderately agree. I can repeat the scale for you at any point.

16. I am always confident of when my next antenatal care visit is
17. The waiting time does cause me problems
18. The health staff take my questions and concerns seriously
19. I am well informed about the purpose of the tests the health staff run
20. I am well informed of when (gestational age) to do the tests
21. I would recommend the services to a friend
22. I would come back if I become pregnant again
23. I am satisfied with the antenatal care service I have received

Worries and concerns (13-item Cambridge Worry Scale (Green 2003))

Most of us worry about something. This list is not meant to give you more things to worry about, but we would just like to know if any of these things are worrying you at all. I will present the statements, and would like to hear how much of a worry it is to you at the moment, from 0 if not a worry to 5 if it is something that you are extremely worried about.

24. Your housing
25. Money problems
26. Your relationship with your husband/partner
27. Your relationship with your family and friends
28. Your own health
29. The health of someone close to you
30. Employment problems
31. The possibility of something being wrong with the baby
32. The possibility of stillbirth
33. Going to hospital
34. Internal examinations (gynecological examination)
35. Giving birth
36. Coping with the new baby

Thank you for your time and cooperation. Good luck with the rest of your pregnancy and the delivery. I wish you all the best!

(Respond to any other questions she may have. If questions arise about how she can find the results of this research, it will be made available on the PNIPH website.)
